# Untargeted Metabolomics Analysis of *Crocus cancellatus* subsp. *damascenus* (Herb.) B. Mathew Stigmas and Their Anticarcinogenic Effect on Breast Cancer Cells

**DOI:** 10.1155/2022/3861783

**Published:** 2022-08-16

**Authors:** Raheleh Shakeri, Bahram Savari, Mahsa N. Sheikholeslami, Tayebeh Radjabian, Jalal Khorshidi, Maliheh Safavi

**Affiliations:** ^1^Department of Biological Science and Biotechnology, Faculty of Science, University of Kurdistan, Sanandaj, Iran; ^2^Center of Excellence in Electrochemistry, Faculty of Chemistry, University of Tehran, Tehran 1417614418, Iran; ^3^Immunoregulation Research Center, Shahed University, Tehran, Iran; ^4^Department of Horticultural Science and Engineering, Research Center of Medicinal Plants Breeding and Development, University of Kurdistan, Sanandaj, Iran; ^5^Department of Biotechnology, Iranian Research Organization for Science and Technology, Tehran 13353-5111, Iran

## Abstract

Safranal, crocin, crocetin, and picrocrocin are major known compounds in the stigma extract of *Crocus sativus* with various medicinal properties. *Crocus cancellatus* is another *Crocus* species that grows extensively in Iran's various regions, such as the Kurdistan province. The predominant metabolites and biological properties of *C*. *cancellatus* have not yet been investigated. The ingredients of the stigma ethanol extract of *C. cancellatus* were investigated using gas chromatography-mass spectrometry (GC-MS) and liquid chromatography with tandem mass spectrometry (LC-MS). The ROIMCR approach was performed to analyze the LC-MS full scan data sets. This method searches the MS regions of interest (ROI) data in the *m*/*z* domain and analyses the results using the multivariate curve-resolution alternating least squares (MCR-ALS) chemometrics technique for simultaneous resolution of two extracts. Also, the antiproliferative properties of *C. cancellatus* against MDA-MB-231 and MCF-7 cancer cells were examined by MTT, dual acridine orange/ethidium bromide test, Annexin V-FITC/PI, and zymography. The GC-MS and LC-MS untargeted metabolomics data analysis of the extract indicated the presence of cytotoxic agents including safranal, crocin, picrocrocin, and crocetin in the stigma ethanol extract of *C. cancellatus*. Biological tests showed that the viability of MDA-MB-231 and MCF-7 cancer cells is decreased following *C. cancellatus* treatment in a time- and dose-dependent way in both monolayer and 3D cell cultures. The MCF-7 cell spheroids had greater resistance to the cytotoxic activity of the extract in 3D cell culture than the MDA-MB-231 cell spheroids. The morphological changes of the cells treated with *C. cancellatus* stigmas extract were indicative of apoptosis. Zymography analysis revealed a similar trend of matrix metallopeptidase-2 (MMP-2) and matrix metallopeptidase-9 (MMP-9) activity in the treated cells with *C. cancellatus* extract in comparison with doxorubicin treatment as a positive control. The findings of this research indicate that the ethanolic extract of *C. cancellatus* stigmas was a good source of bioactive metabolites with anticancer activity.

## 1. Introduction

Over 80 species of the genus *Crocus* L. have been identified worldwide [1]. The most common species of this genus is *Crocus sativus*. Saffron, the most costly food colorant and flavor in the world, is derived from the dried stigmas of *C. sativus,* and it has been used in folk medicine to treat a range of disorders since ancient times [1–4]. Previous research has uncovered important information about the anti-cancer properties of saffron and its constituents [5]. However, there are no significant findings regarding the underlying compounds and anticancer characteristics of another *Crocus* species known as *Crocus cancellatus* subsp. *damascenus* [1]. The main bioactive constituents present in saffron are crocetin, crocin, safranal, and picrocrocin. Crocetin and crocin (a diester of crocetin with gentiobiose) are carotenoid compounds that have been shown in cell culture systems and animal models to be efficient at preventing or treating cancer. Safranal is a cyclic terpenic aldehyde obtained from the enzymatic and thermal degradation of picrocrocin and zeaxanthin. Picrocrocin is a monoterpene glycoside [6, 7]. *Crocus cancellatus* is abundant throughout Iran, especially in the province of Kurdistan [1]. The main phytochemicals and the cytotoxic properties of stigmas of this species have not been studied yet.

Chemometric methods can be used to analyze complex data in chemical and biological systems. Recently, a novel chemometrics method based on the combination of the region of interest (ROI) [8–11] and the multivariate curve resolution-alternating least squares (MCR-ALS) method [12–14] has been employed for filtering, compressing, and resolving the complex data sets that are gathered by LC-MS analysis. The ROI method reduces the raw MS data without considerable loss of helpful information or mass accuracy of the measured data by searching within the region of interest for the *m*/*z* domain [15, 16]. The ROI method compresses a large amount of big complex data [9, 10]. In the current study, ROI-MCR-ALS methodology based on LC-MS full scan data sets was performed to resolve and identify many compounds in *C. cancellatus* stigmas compared to *C. sativus*. This research aimed to characterize the contents of the stigma ethanol extract of *C. cancellatus* and determine their effect on the growth of MDA-MB-231 and MCF-7 human breast cancer cells.

## 2. Materials and Methods

### 2.1. Chemicals and Materials

The cell culture medium (RPMI 1640), fetal bovine serum (FBS), and penicillin–streptomycin were purchased from GibcoBRL (Life Technologies, Scotland). The culture plates were obtained from Nunc (Roskilde, Denmark). Dimethyl sulfoxide (DMSO) and ethanol were obtained from Merck (Germany). 3-(4, 5-dimethylthiazol-2-yl)-2, 5-diphenyltetrazolium bromide (MTT) and phosphate buffer saline (PBS) tablets were obtained from Sigma-Aldrich (USA). The Annexin V-FITC/Propidium iodide kit was obtained from MabTag's Company, Germany. The MDA-MB-231 (a highly invasive human breast cancer cell line) and MCF-7 (human breast adenocarcinoma cell line) were purchased from the Pasteur Institute of Iran (Tehran).

### 2.2. Plant Collection and Extraction

The plant of *C*. *cancellatus* subsp. *damascenus* (voucher specimen number: 725) was collected in autumn 2019 from the areas between latitude 35°18′36″N and longitude 46°57′26″E at an elevation of 1916 meters above sea level in Sanandaj, Kurdistan province, Iran. It was authenticated at the Research Center of Agricultural and Natural Resources of Sanandaj, Kurdistan province, Iran by Hosein Maroofi. The stigmas of the plants were separated and dried in the shade, powdered, and macerated with ethanol for 24 h in a dark place at a powder: solvent ratio of 1 g : 100 mL at room temperature. The extract was collected and filtered. The same material was subsequently extracted for the second and third time. Consequently, the ethanol solvent in the collected extract was concentrated using rotary evaporation. The concentrated extract was dried to determine the exact dry weight [17].

### 2.3. Gas Chromatography-Mass Spectrometry (GC-MS) Analysis

GC/MS analysis of the extracts was carried out using an Agilent 6890 series gas chromatograph equipped with a column VARIAN, cp-sil 8cb-ms (50 m length, 0.25 mm inner diameter, 0.25 m film thickness) coupled with an Agilent 5973 Network, mass selective detector. The moving phase was 99.999 percent helium with a steady flow rate of 1 mL/min via the column. The temperature was set at 60°C, ascending to 280°C, and the injector temperature was 280°C. The injector volume was 1 *μ*L. A 70 eV and the electron beam was used to generate ions. Masses between *m*/*z* 40 and 600 were identified, and the acceleration voltage was turned on after a 4-minute solvent delay. The ingredients of the extracts were recognized by computing retention times and fragmentation patterns of the related peak compared to those of the NIST/Wiley internal reference mass spectrum library.

### 2.4. Liquid Chromatography with Mass Spectrometry (LC-MS)

The separation was carried out chromatographically on an Atlantis T3-C18 analytical column (3.0 m, 2.1 100 mm). At a flow rate of 0.15 mL/min, the column was eluted with a mobile phase composed of 95% methanol and 5% water containing 0.1 percent formic acid. The column was kept at a temperature of 45°C. The injection volume was 10 *μ*L, and the analysis duration was 30 min. Electrospray ionization (ESI) was used in conjunction with a triple quadrupole mass analyzer in the positive mode, with the spray voltage set at 4500 V. The nebulizer gas was nitrogen (N2 Garde 5), and the nebulizer pressure was adjusted to 40 psi with a source temperature of 120°C. The collision energy was 35 eV.

### 2.5. Chemometric Data Analysis

In this work, the ROIMCR method combining the regions of interest and Multivariate Curve Resolution-Alternating Least Squares (MCR-ALS) methods has been used [8]. This method searches the regions of interest in the LC-MS full scan data (MS-ROI) that is applied for compressing and filtering data without losing essential information on mass accuracy and spectral resolution. All the chemometrics analyses were carried out in MATLAB 2016b [8, 9, 12].

#### 2.5.1. Region of Interest (ROI)

Compressing a large amount of data without reducing the information is a significant step in data analysis. In the present work, the region of interest (ROI) has been performed for filtering and compressing the LC-MS full-scan data without losing significant information on data resolution and the mass accuracy in the *m*/*z* domain [8, 9]. The ROI method was used to create a data matrix suitable for multivariate modeling. This matrix contains the mass traces as well as the intensities of the same *m*/*z* values of distinguished dimensions [8, 9, 18]. Three parameters have a significant effect on the resulting mass intensity matrix: (1) the threshold of the mass intensity (SNRthr), (2) the mass accuracy or *m*/*z* error of the spectrometer, and (3) the minimum number of retention times that must be included in an ROI. The mentioned parameters in this study were 0.1% of the maximum MS signal intensity measured on each sample, 1 Da/e, and 50, respectively.

The output parameters of this algorithm are a vector including the average of all *m*/*z* data from the same ROI, a matrix of data comprising MS spectra at each retention time scan (MS-ROI), and a cell array that contains the data regarding the *m*/*z* values, retention time, mass intensities, scan numbers, and average *m*/*z* values of ROIs. Finally, to be appropriate for MCR-ALS analysis, all ROI *m*/*z* values with significant traces were organized in the MSROI data matrices [8].

#### 2.5.2. MCR-ALS Analysis

Using a bilinear decomposition approach, MCR-ALS can resolve the profiles (spectra, concentration, pH profiles, time profiles, elution profiles, etc.) of the components in mixtures without providing any information regarding their compositions. This algorithm decomposed the instrumental response matrix (*D*) to the *C* matrix (concentration/elution profiles) and the *S*^*T*^ matrix (pure spectra) of the components present in a mixture (equation (1)). The residual and nonmodeled information is included in the matrix, and the symbol *T* indicates a transposed matrix:

(1) $$\begin{array}{c} D=C.S^{T}+E. \end{array}$$

**Figure 1:**
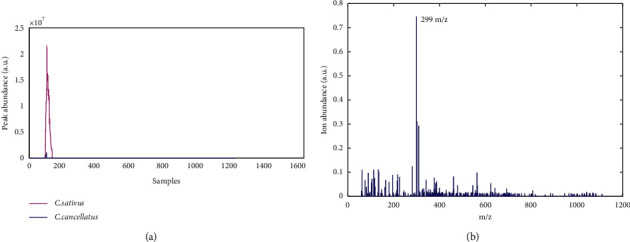
An example of MCR-ALS outcomes after MSROI data matrix analysis of *C. sativus* and *C. cancellatus*. (a) The elution profiles of two samples were resolved by the MCR-ALS. (b) The most abundant ion in this component's mass spectrum, obtained from MCR-ALS, is *m*/*z* 299.

In the first step of the MCR-ALS method, the purest spectra derived by the SIMPLISMA (SIMPLe-to-use Interactive Self-modeling Mixture Analysis) algorithm served as the initial estimations for singular value decomposition (SVD) analysis. Then, suitable constraints must be applied to reduce the ambiguities associated with the resolution of the *D* matrix. In this study, nonnegativity and unimodality constraints were performed to optimize ALS for elution and mass spectra profiles. Also, 60 components containing one elution and mass spectra profiles were resolved by MCR-ALS [12, 19]. An example of the resolution of one of the components is shown in Figure 1. The peak area and heights of each elution profile retrieved by MCR-ALS can be related to the concentration of components. Finally, the spectral *m*/*z* mass values for each spectrum and their elution profiles recovered by MCR-ALS were performed to identify possible chemical compounds in the samples.

#### 2.5.3. Software

MATLAB (The Mathworks Inc., https://www.mathworks.com) was used to perform MCR-ALS using the MCR-ALS toolbox, which is openly accessible from http://www.mcrals.info [14].

The MATLAB functions for the ROI process can be found at: https://cidtransfer.cid.csic.es/descarga.php?enlace1=298348e5b34daf9e844835352bafa6%2045250ee1.

Further, a detailed description of the ROIMCR procedure's use is also provided on this page. https://www.nature.com/protocolexchange/protocols/4347 [8, 9].

### 2.6. MTT Assay

The MTT test was used to evaluate the cytotoxic potential of the extracts against cancer cells [20]. In 96-well plates, 15,000 cells were distributed in 200 *µ*l of growth media and incubated with different concentrations of the extract for 48 and 72 hours in an incubator at 37°C under 5% CO_2_ pressure after adhering to the bottom of the plate and taking morphology. To quantify the number of living and dead cells in each well, the culture medium was drained, and after being washed with PBS, the MTT solution (0.5 mg/mL) was added. The plate was incubated for four hours at 37°C with 5% carbon dioxide pressure. The viable cells transform the MTT solution into formazan crystals. To dissolve formazan crystals, the wells were depleted of the solution, and 100 mL of DMSO was added. As soon as the crystals were completely dissolved in DMSO, the 570 nm absorbance of each well was measured by a microplate reader (BioTek, USA). To determine the IC_50_ value, first, the inhibition percentage of each extract was calculated; next, the IC_50_ value (concentration equivalent to 50 percent survival of cancer cells) was obtained for each extract using regression analysis of the dose-response curve.

### 2.7. Fluorescence Labeling of Cells with Acridine Orange (AO) and Ethidium Bromide (EB)

The cancer cells were incubated with the corresponding IC_50_ values of the extract for 12 h. Following trypsinization and washing with PBS, the cells were mixed with 100 *μ*L of acridine orange and ethidium bromide (1 : 100 mg/mL) solution and observed under a fluorescent microscope (Zeiss, Germany) [21]. Green-colored cells are considered viable, while red/yellow-colored cells are considered dead. For quantitative measurement, a minimum of 200 cells were counted in different fields under a fluorescence microscope. The percentage of cells undergoing apoptosis was calculated according to the following equation:

(2) $$\begin{array}{c} \text{The percentage of cells undergoing apoptosis}=\left[\frac{\text{Total apoptotic cells}}{\text{Total cells counted}}\right]\times 100\text{.} \end{array}$$

### 2.8. Flow Cytometric Analysis of Cells with Apoptotic Markers

One of the markers of apoptotic cells is the translocation of phosphatidylserine from inside the cell membrane to the exterior, which can be detected by staining cells with Annexin V-FITC. Propidium iodide is another fluorescent dye used to identify necrotic and late apoptotic cells. For this analysis, the cancer cells were cultured with the IC_50_ values of the extract. After 48 hours, the cells were trypsinized, washed using PBS, and resuspended in the binding buffer. 5 *µ*L of each fluorescent dye were added to the cell suspension, kept in the dark at room temperature for 15 minutes, and analyzed using flow cytometry (Partec PAS, Germany) without delay.

### 2.9. Gelatin Zymography

Gelatin zymography is one of the effective methods for measuring MMP-2 (EC 3.4.24.24) and MMP-9 (EC 3.4.24.35) activities in cells and growth medium [22]. In order to perform the analysis, the medium from the cells treated with extract without serum was sampled, and the Bradford method was used to measure the total protein concentrations [23]. 20 *μ*g of each sample was mixed with 5X nonreducing sample buffer (including 125 mM Tris HCl (pH 6.8), 20% glycerol, 4% SDS, and 0.01% bromophenol blue) and was loaded into each well of 10% acrylamide gel containing gelatin. The gel was run at 100 V, and after band separation, it was separated from glass and washed for 2 × 30 min with washing buffer (50 mM Tris HCl (pH 7.5), 1 *μ*M ZnCl_2_, 5 mM CaCl_2_, and 2.5% Triton X-100) at room temperature with agitation. After washing, the gel was immersed in incubation buffer (50 mM Tris HCl (pH 7.5), 5 mM CaCl_2_, 1% Triton X-100, 1 *μ*M ZnCl_2_) for 5–10 min at 37°C with shaking. Then, the gel was incubated in a fresh incubation buffer for 48 h, stained with staining solution (40 mL methanol, 10 mL acetic acid, 0.5 g Coomassie Blue, and 50 mL H_2_O), and incubated with destaining solution (10 mL acetic acid, 40 mL methanol, and 50 mL H_2_O), respectively. The white bands on a uniformly blue background of gel are indicative of MMP activity. ImageJ was used to analyze the gel bands.

### 2.10. Statistical Analysis

Three independent experiments were used to obtain the data. The results are reported as the mean standard deviation. A *t*-test was conducted independently to analyze the data using the SPSS software (SPSS Inc., Chicago, IL, USA). The difference between the two groups was considered significant at a *p* value <0.05.

## 3. Results

### 3.1. GC-MS Analysis of the Stigma Ethanolic Extract *C. cancellatus*

GC-MS analysis revealed the occurrence of safranal (retention time: 18.09 min) in the ethanolic extract of *C*. *cancellatus.* The list of the components identified in the ethanolic extract of *C. cancellatus* stigmas by GC-MS is presented in Table 1 (The chromatogram is shown in Figure S1, supplemental file).

### 3.2. MCR-ALS of Full Scan LC-MS Chromatograms

The MCR-ALS technique was applied to analyze the LC-MS data sets of the *C. sativus* and *C. cancellatus* extracts in the positive and negative MS ionization modes (see Figure 1). The profiles of elution for 19 components have been shown in Figure 1(a). Regarding the peak area of this component in both samples, it is clear that the concentration of this compound for samples of *C. sativus* and *C. cancellatus* is different.  Figure 1(b) shows the retrieved mass spectrum of this ingredient using the MCR-ALS. Upon examination of the ion with the highest abundance at *m*/*z* (299), this component has been identified as an important component in the *C. sativus* and *C. cancellatus* samples. The proportion of this compound in the sample of *C. sativus* is much higher than in the sample of *C. cancellatus.* MCR-ALS identified all 60 components using a similar approach. Finally, 24 and 14 common metabolites for *C. sativus* and *C. cancellatus* samples, respectively, were identified in both positive and negative ionization modes.  Table 2 shows the components retrieved by the MCR-ALS method for *C. sativus* and *C. cancellatus*. In this table, the name of the compounds, measured mass, identified adducts, retention times, and peak area for samples of *C. sativus* and *C. cancellatus* are given.

### 3.3. Cytotoxicity Evaluation of the Stigma Ethanolic Extract of *C. cancellatus* on MDA-MB-231 and MCF-7 Cancer Cells

**Figure 2:**
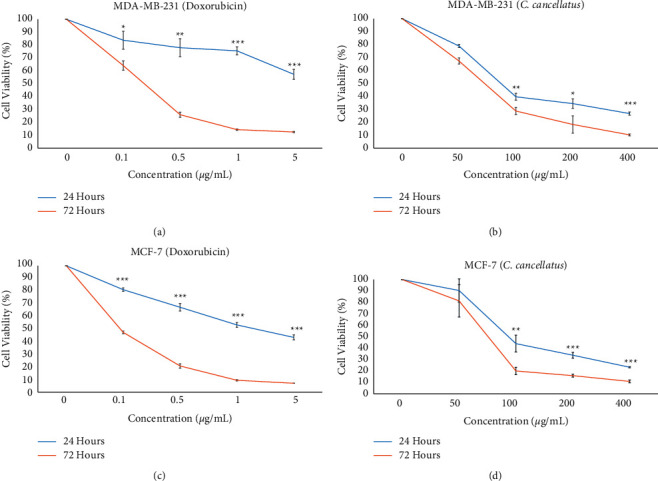
Viability of monolayer cultured breast cancer cells treated to different concentrations of doxorubicin and stigmas ethanol extract of *C. cancellatus* subsp. damascenus. Survival of doxorubicin- (a) and *C. cancellatus*-treated (b) MDA-MB-231 cells. Survival of doxorubicin- (c) and *C. cancellatus*-treated (d) MCF-7 cells. Data are mean of three independent replicates ± SD; ^*∗∗∗*^*P* < 0.001, ^*∗∗*^*P* < 0.01, and ^*∗*^*P* < 0.05 for the significant differences between 24 and 72 h incubation.

MTT was employed to evaluate the influence of stigma ethanol extract of *C. cancellatus* for 24 and 72 hours on the viability of MDA-MB-231 and MCF-7 cancer cells. Based on the results, *C. cancellatus* represented cytotoxicity toward cancer cell lines (Figure 2). For MDA-MB-231, the IC_50_ values (Table 3) of *C. cancellatus* were 97.54 ± 9.67 and 69.06 ± 6.55 *μ*g/mL following 24 and 72 hours of incubation, respectively. The extract's IC_50_ values (Table 3) on MCF-7 cells were 131.44 + 8.35 *μ*g/mL for 24 hours and 78.70 + 9.09 *μ*g/mL for 72 hours of treatment. Decreasing IC_50_ values over time indicates the time-dependent effect of the extract. Both MDA-MB-231 and MCF-7 were equally susceptible to the effects of the extracts. Doxorubicin was employed as a positive control.

### 3.4. Cytotoxicity Evaluation of the Stigma Ethanolic Extract of *C. cancellatus* on MDA-MB-231 and MCF-7 Cell Spheroids

**Figure 3:**
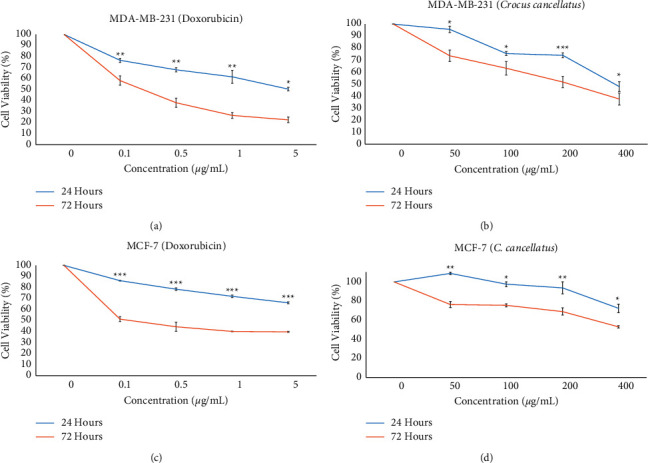
Viability of breast cancer cell spheroids exposed to various concentrations of doxorubicin and stigma ethanol extract of *C. cancellatus* subsp. damascenus. Survival of doxorubicin- (a) and *C. cancellatus*-treated (b) MDA-MB-231 cells. Survival of doxorubicin- (c) and *C. cancellatus*-treated (d) MCF-7 cells. Data are mean of three independent replicates ± SD; ^*∗∗∗*^*P* < 0.001, ^*∗∗*^*P* < 0.01, and ^*∗*^*P* < 0.05 for the significant differences between 24 and 72 h incubation.

Tumor cells generally exhibit less sensitivity to chemotherapeutic agents in 3D cell culture models than those in monolayer culture [24]. Cell viability of MDA-MB-231 and MCF-7 spheroids following exposure to the various concentrations of stigma ethanolic extract of *C. cancellatus* was evaluated by the MTT test. As shown in Figure 3, spheroids' viability declined as a function of dosage and time compared to untreated spheroids. The extract's IC_50_ value (Table 3) of the extract against MDA-MB-231 spheroids was approximately 2-3 times higher than the corresponding value in monolayer culture. MCF-7 spheroids had more resistance to the extract in comparison to MCF-7 in monolayer cell culture.

### 3.5. The Ability of the Stigma Ethanolic Extract of *C. cancellatus* to Elicit Apoptosis on MDA-MB-231 and MCF-7 Cells

**Figure 4:**
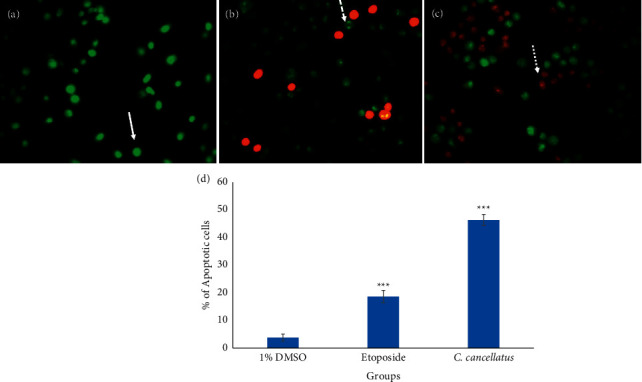
Fluorescence labeling of MDA-MB-231 cells with acridine orange and ethidium bromide. The monolayer cultured cells after treatment with (a) DMSO 1% as control, (b) etoposide at a concentration equal to the IC_50_ as a positive control, and (c) stigma ethanol extract of *C. cancellatus* subsp. *damascenus* at a concentration equal to the IC_50_. Live cells are shown by the solid arrow; dashed and dotted arrows denote cells that are in the early and late stages of apoptosis, respectively. Fluorescence microscope images were captured at 400x magnification. (d) Percentage of cells that have undergone apoptosis. Data are shown as the mean and standard error of the mean based on three data points. ^*∗∗∗*^*P* < 0.001 compared to control.

**Figure 5:**
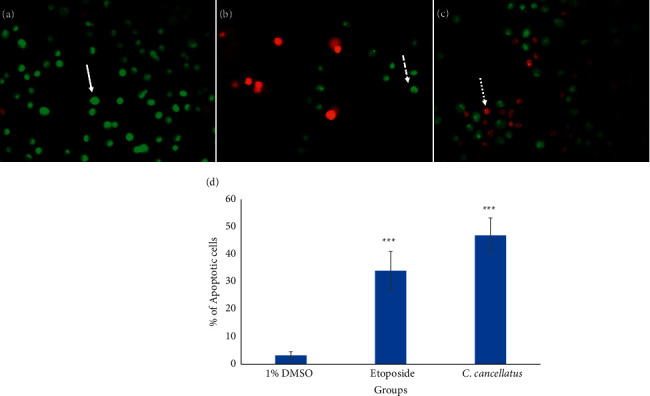
Fluorescence labeling of MCF-7 cells with acridine orange and ethidium bromide. The monolayer cultured cells after treatment with (a) DMSO 1% as control, (b) etoposide at a concentration equal to the IC_50_ as a positive control, and (c) stigma ethanol extract of *C. cancellatus* subsp. *damascenus* at a concentration equal to the IC_50_. Live cells are shown by the solid arrow; dashed and dotted arrows denote cells that are in the early and late stages of apoptosis, respectively. Fluorescence microscope images were captured at 400x magnification. (d) Percentage of cells that have undergone apoptosis. Data are shown as the mean and standard error of the mean based on three data points. ^*∗∗∗*^*P* < 0.001 compared to control.

**Figure 6:**
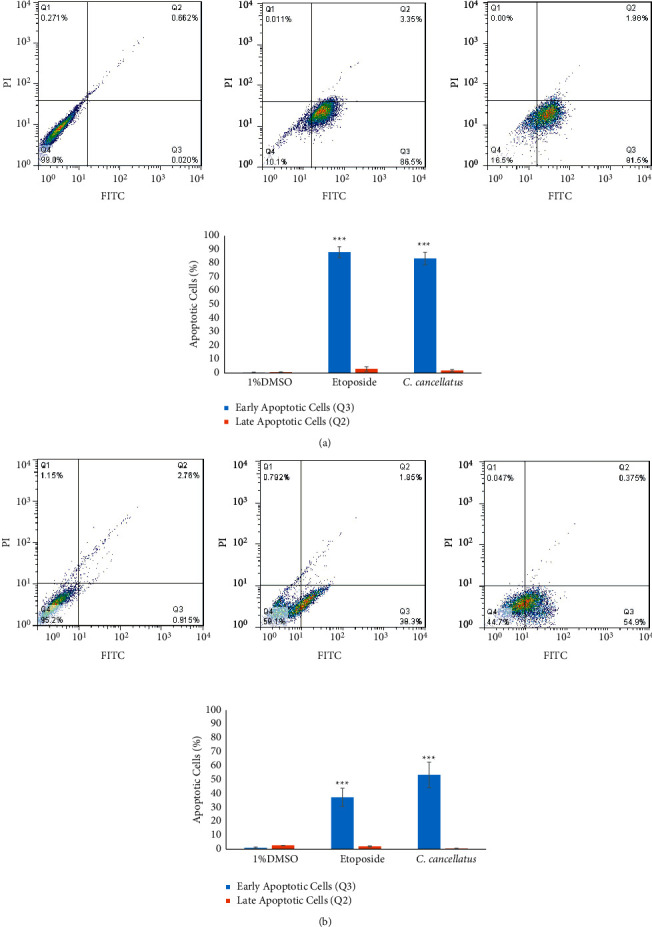
Quantifying cell death by labeling MDA-MB-231 (a) and MCF-7 (b) cells with annexin V-FITC/PI using flow cytometry. From left to right, 48 h treatment with DMSO 1% (negative control), etoposide (positive control), and stigma ethanol extract of *C. cancellatus* subsp. *damascenus*. Statistical analysis of flow cytometry results was performed with three independent experiments. ^*∗∗∗*^*P* < 0.001 compared to control (DMSO 1%).

Apoptosis-associated changes of cell membranes in the cancer cells during incubation with the stigmas ethanolic extract of *C. cancellatus* and etoposide (as a positive control) were assessed by labeling cells with acridine orange (AO) and ethidium bromide (EB) fluorescent agents. The finding indicated that the extract destroys cancer cells by promoting them to undergo apoptosis. Based on Figures 4 and 5, live cells appear green in color as a result of the acridine orange penetration, whereas apoptotic cells are orange-red in color due to ethidium bromide penetration. The induction of apoptosis was also examined by staining the treated cells with two fluorescent agents, Annexin V-FITC and PI. The number of stained cells was counted using flow cytometry. The flow cytometry analysis was conducted after both cell lines were incubated for 48 hours with the corresponding IC_50_ values of either the extract or etoposide (as a positive control). As shown in Figure 6, unstained living cells are in the Q4 region. Apoptotic and necrotic cells are stained with Annexin V-FITC and PI, respectively. Thus, Q3 (Annexin V-FITC^+^/PI^−^) and Q2 (Annexin V-FITC^+^/PI^+^) correspond to early apoptotic and late apoptotic cells, respectively. Results exhibited that 81.5% of MDA-MB-231 and 54.9% of MCF-7 cells are in the early stages of apoptosis following treatment with the stigma extract of *C. cancellatus*.

### 3.6. Gelatinase Activity of MDA-MB-231 and MCF-7 Cells Exposed to the Ethanolic Extract of *C. cancellatus* Stigma

**Figure 7:**
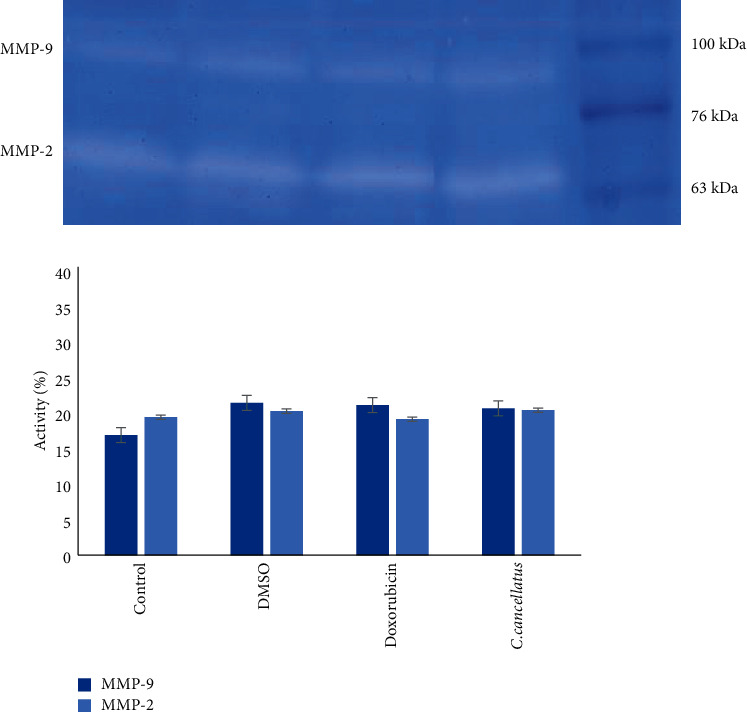
The activity of MMP-9 and MMP-2 in MDA-MB-231 cells. Following treatment of cells with DMSO 1% as a control, doxorubicin as a positive control, and stigmas ethanol extract of *C. cancellatus* subsp. *damascenus*, the extracellular medium was employed to investigate MMP-9 and MMP-2 activities by gelatin zymography. Quantification of each band is expressed as mean ± standard error of mean (*n* = 3). No significant difference was observed.

**Figure 8:**
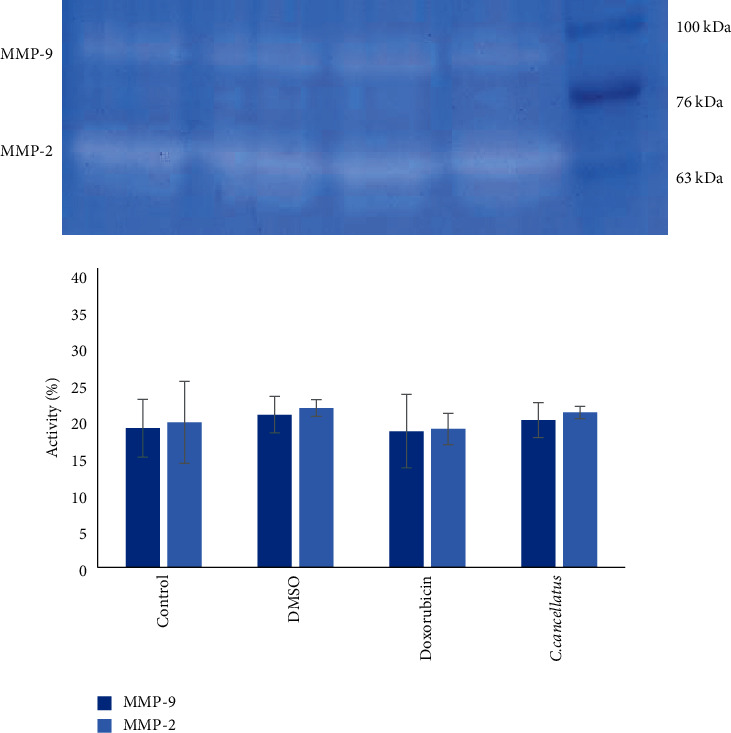
The activity of MMP-9 and MMP-2 in MCF-7 cells. Following treatment of cells with DMSO 1% as a control, doxorubicin as a positive control, and stigmas ethanol extract of *C. cancellatus* subsp. *damascenus*, the extracellular medium was employed to investigate MMP-9 and MMP-2 activities by gelatin zymography. Quantification of each band is expressed as mean ± standard error of mean (*n* = 3). No significant difference was observed.

The effect of stigmas ethanolic extract of *C. cancellatus* on the activity of MMP-2 and MMP-9 in the growth media of MDA-MB-231 and MCF-7 were investigated using the gelatin zymography method. Data revealed that MMP-2 and MMP-9 activity in both cell lines' growth media containing the extract was similar to those of enzymes in the existence of doxorubicin (Figures 7 and 8).

## 4. Discussion

Over the last decades, there has been a trend to find new sources of natural anticancer. Due to the side effects of synthetic anticancer agents, natural product-based drugs have been becoming more popular and have resulted in an increased interest in the search for chemotherapeutic agents in natural sources [25]. The dried stigmas of *C. sativus* (known as saffron) contain various anticancer agents, including safranal, crocin, picrocrocin, and crocetin [2]. *C. cancellatus*, a herbaceous plant of the family Iridaceae, is native to different areas of Iran, especially in Kurdistan province; however, no investigation has evaluated the main phytochemicals and the cytotoxic properties of stigmas of *C. cancellatus* [26]. Gas chromatography-mass spectrometry (GC-MS) and liquid chromatography with tandem mass spectrometry (LC-MS) were used to determine the ingredients of the stigma ethanol extract of *C. cancellatus*. Chemometric analysis of the LC-MS full scan data was performed using the ROIMCR approach, which selects MS regions of interest (ROI) from full-scanning data sets and analyses them by the Multivariate Curve-Resolution Alternating Least Squares (MCR-ALS) chemometrics method for simultaneous resolution of the extract in comparison with *C. sativus* extract. Based on the most abundant ion at *m*/*z* (299), safranal was identified as an important compound in the *C. sativus* and *C. cancellatus* samples. In total, 24 compounds were detected in negative ionization mode, among which 14 compounds were identified as ferulic acid, 3, 5, 5-trimethyl-4-hydroxy-1-cyclohexanon-2-ene, safranal, *gamma*-crocetin, nonanoate, carotene, crocusatin G, (all-*E*)-crocetin, picrocrocin, 5-hydroxymethyl-2-furancarboxaldehyde, rhamnetin, *beta*-Sitosterol, norathyriol, and riboflavin. In positive ionization mode, 14 compounds were detected in both samples, among which 9 compounds were identified as isopimpinellin, 4H-pyran-4-one-2, 3-dihydro-3, 5-dihydroxy-6-methyl, Osthol, 3, 5, 5-trimethyl-2-hydroxy-1, 4-cyclohexadion-2-ene, 2, 4, 4-trimethyl-3-formyl-6-hydroxy-2, 5-cyclohexadiene-1-one, Methyl arachidate, kaempferol 3-O-alpha-L-rhamnofuranoside, isophorone, and crocin 4. In saffron, the four most important bioactive substances are crocin, crocetin, picrocrocin, and safranal. Saffron's color, aroma, and flavor are determined by the crocin, safranal, and picrocrocin, respectively. Picrocrocin is a monoterpene glycoside, and the isolation of sugars from picrocrocin yields safranal, the essential oil of saffron. Glucosidic derivatives of crocetin are called crocin and are categorized according to the number of attached sugars [27, 28]. Crocin 4 is a monoglucosyl ester of crocetin that was identified in *C. cancellatus* at *m*/*z* (169) and a retention time of 15.40 minutes. 3, 5, 5-Trimethyl-4-hydroxy-1-cyclohexanon-2-ene, 2, 4, 4-trimethyl-3-formyl-6-hydroxy-2, 5-cyclohexadien-1-one, and isophorone are 6-membered ring compounds which have been previously reported as volatile constituents of saffron [29]. Kaempferol 3-O-alpha-L-rhamnofuranoside is a type of flavonoid found in herbs and spices [30]. It was determined through GC-MS analysis that the main compounds in the ethanolic stigmas extract of *C. cancellatus* are fatty acids (lauric acid, octanoic anhydride, myristic acid, pentadecanoic acid, palmitic acid, palmitic acid ethyl ester, margaric acid, linoleic acid, and stearic acid). Furthermore, GC-MS analysis revealed that the extract contained safranal. GC-MS analysis of the *C. cancellatus* extract by Loizzo et al. revealed that the most abundant compounds are 5-(hydroxymethyl)-2-furancarboxaldehyde, methyl oleate, methyl stearate, and osthol [31].

In recent years, *C. sativus* stigmas have been widely researched for their potential effects on cancer and other diseases [4, 32]. According to the identification of the main bioactive constituents of saffron, the antiproliferative effect of *C. cancellatus* stigmas extract in both 2D monolayer and 3D cell cultures of MDA-MB-231 and MCF-7 human breast cancer cells were examined for the first time. Data showed that ethanolic stigma extract of *C. cancellatus* inhibits proliferation of both MDA-MB-231 and MCF-7 cancer cells in 2D monolayer cell culture in a way that is dependent on both concentration and time. The inhibitory effect of *C. cancellatus* on MCF-7 was similar to that on MDA-MB-231 cells after 24 and 72 h of treatment. Since MCF-7 epithelial cells are estrogen-dependent (ER^+^: estrogen receptor-positive) and of low metastatic potential, MDA-MB-231 cells are estrogen-independent (ER^–^) and have high invasiveness [33], cytotoxicity results indicated that the antiproliferative activity of *C. cancellatus* is not dependent on the existence of estrogen receptors. This is the first report on the cytotoxic potential of *C. cancellatus* stigmas. The cytotoxicity effect of stigmas ethanolic extract on both breast cancer cells in spheroids was also validated. Spheroids are excellent models for tumor studying and cytotoxic agent screening [34]. *C. cancellatus* also exhibited cytotoxic activity against MDA-MB-231 and MCF-7 spheroids in a concentration- and time-dependent manner. Our findings suggest that spheroids are less susceptible to the cytotoxic activity of the extract than 2D cultures. Unlike 2D culture, the resistance of MCF-7 spheroids to the toxic effect of the extract was higher than that of MDA-MB-231 spheroids.

One of the major ways anticancer drugs suppress cancer cells is the induction of apoptosis [35]. The results of the AO/EB double staining assay displayed that the morphological changes observed in cells treated with *C. cancellatus* are attributed to apoptosis. Apoptosis is distinguished from other types of cell death by specific morphological changes such as shrinkage of nuclei and cells, blebbing of the plasma membrane, and fragmentation of oligo-nucleosomal DNA [36]. Unlike apoptosis, loss of cell membrane integrity is a typical feature of necrosis. The accompanying cell membrane changes of apoptosis can be differentiated from necrosis by the Dual AO/EB fluorescent staining test. AO can pass through the intact cell membrane, bind to DNA, and emit green fluorescence. EB has only been permitted to cross damaged membranes and emit orange-red fluorescence upon binding to DNA. So, cells that are in the early stages of apoptosis, as well as viable cells, appear green, while those in the late stages of apoptosis, as well as necrotic cells, appear orange-red. Early and late apoptotic cells exhibit bright green and orange fluorescent spots within the nuclei, respectively, as a result of nuclear fragmentation and chromatin condensation; these phenomena do not occur in the nuclei of necrotic cells [37]. The stages of apoptosis and cell death in single-cell suspensions were analyzed by Annexin-V-FITC and propidium iodide (PI). A unique biochemical event in apoptotic cells is the translocation of phosphatidylserine (PS) from the inner side of the cytoplasmic membrane to the outer surface of the cell membrane, a process that Annexin-V-FITC can monitor. During this reaction, Annexin-V-FITC is specifically bound to PS and detects early apoptotic cells. As PI (an intercalating agent) is unable to penetrate healthy cell membranes, cells in the late stages of apoptosis and necrotic cells are stained by it due to the loss of cell membrane integrity [38]. Annexin V-FITC/PI staining exhibited that stigma ethanol extract of *C. cancellatus* promoted early apoptosis in MDA-MB-231 and MCF-7 cells after 48 h of incubation.

It has been shown that overexpression of matrix metalloproteinases (MMPs) by tumor and stromal cells has been linked to cell migration and metastasis [39]. MMPs are secreted and zinc-dependent endoproteinases that break down extracellular matrix (ECM) proteins (collagen, gelatin, fibronectin, vitronectin, and laminin) [40]. On the basis of favored substrates, MMPs are initially categorized into collagenases (MMP-1, MMP-8, MMP-13), gelatinases (MMP-2, MMP-9), stromelysins (MMP-3, MMP-10, MMP-11), matrilysins (MMP-7), and membrane-associated MMPs (MT1-MMP/MMP-14, MMP-15, MMP-16, MMP-17, MMP-24, MMP-25). Among MMPs, MMP-2 and MMP-9 are involved in breast tumor invasion [41]. In an attempt to study the antimetastatic potential of the stigmas ethanol extract of *C. cancellatus*, the zymography method was used to determine the activity of MMP-2 and MMP-9 in the ECM of treated cells. The findings suggest that the behavior of MMP-2 and MMP-9 in the treated cells with the extract is similar to that of the treated cells with doxorubicin as a positive control.

## 5. Conclusion

This work has shown that stigma ethanolic extract of *C. cancellatus* inhibited the proliferation of MDA-MB-231 and MCF-7 human breast cancer cell lines. The AO/EB double staining assay and Annexin V-FITC/PI validated that the treated cells were undergoing apoptosis. The antimetastatic effect of the stigma ethanol extract of *C. cancellatus* was similar to doxorubicin. Analyses by GC-MS showed safranal to be present in *C. cancellatus* extract. A potent strategy based on selecting the LC-MS regions of interest (ROI) and multivariate curve resolution (MCR-ALS) was carried out to analyze *C. cancellatus* LC-MS data compared to *C. sativus*. The metabolites and compounds present in the *C. sativus* and *C. cancellatus* samples were analyzed, and the concentration differences between the two samples were evaluated. The current investigation provides essential intuitions into the antiproliferative effect of *C. cancellatus,* which may be related to the presence of safranal, crocin, crocetin, and picrocrocin.

## Figures and Tables

**Table 1:** GC-MS analysis of *C. cancellatus* subsp. damascenus stigmas ethanol extract. Safranal is shown in bold type.

Retention time (min)	Component name	% of total
5.25	Hydroxyacetone	0.33
6.16	2-Methyl-2-butenal	0.66
6.30	2-Methylbutyraldehyde	0.23
6.68	Glycolic acid, ethyl ester	0.31
6.80	Propanoic acid, 2-oxo-, methyl ester	0.93
7.78	Furfural	1.7
10.97	5-Methylfurfural	0.65
11.46	2, 3-Dihydro-3, 5-dihydroxy-6-methyl-4H-pyran-4-one	0.7
13.29	*β*-Isophorone	1.12
14.43	Resorcinol, 5-methyl-	0.92
15.79	*α*-Isophorone	0.42
16.42	4-Oxoisophorone	0.76
16.50	2-Hydroxyisophorone	0.49
16.80	2, 3-Dihydro-3, 5-dihydroxy-6-methyl-4H-pyran-4-one	2.2
17.17	2, 2, 6-Trimethyl-1, 4-cyclohexanedione	0.55
18.09	Safranal	0.93
18.80	Eucarvone	3.32
19.00	3, 5, 5-Trimethyl-2-hydroxy-1, 4-cyclohexadione-2-ene	1.45
19.41	2-Furancarboxaldehyde, 5-(hydroxymethyl)	10.22
21.18	Nepetalactone	0.42
21.52	2, 6, 6-Trimethyl-4-oxo-2-cyclohexen-1-carboxaldehyde	14.52
21.75	2-Methoxy-4, 4, 6-trimethylcyclohexa-2, 5-dien-1-one	1.83
23.30	2, 4, 4-Trimethyl-3-carboxaldehyde-5-hydroxy-2, 5-cyclohexadien-1-one	0.58
23.74	Methylcyclohexane	2.4
26.18	2, 4-bis (1,1-Dimethylethyl) phenol	2.14
27.47	Lauric acid	1.17
27.80	Octanoic anhydride	1.51
28.23	D-Allose	3.71
31.97	Myristic acid	0.89
34.05	Pentadecanoic acid	0.21
34.16	Diisobutyl phthalate	0.3
35.20	7, 9-Di-tert-butyl-1-oxaspiro (4, 5) deca-6, 9-diene-2, 8-dione	0.52
36.31	Palmitic acid	14.36
36.61	Palmitic acid, ethyl ester	0.34
37.52	Margaric acid	0.88
39.59	Linoleic acid	8.77
39.71	Linoleic acid	5.47
39.98	Stearic acid	3.04
43.16	4, 8, 12, 16-Tetramethylheptadecan-4-olide	0.25
43.67	Hexanedioic acid, bis (2-ethylhexyl) ester	0.19

**Table 2:** Metabolites in the extract of *C. cancellatus* based on LC-MS resolved by MCR-ALS method in comparison with the extract of *C. sativus*.

Mode	Name^a^	Experimental mass^b^	Adduct^c^	*R*_*t*_^d^	Peak area^e^ (*C.sativus*)	Peak area^e^ (*C.cancellatus*)
(ESI)^−^	Ferulic acid	387	2M-H	1.11	8.51 × 10^6^	1.67 × 10^8^
	Unknown	128		1.17	1.18 × 10^7^	2.61 × 10^8^
	3, 5, 5-Trimethyl-4-hydroxy-1-cyclohexanon-2-ene	134	M-H_2_O-H	1.31	3.52 × 10^6^	3.67 × 10^8^
	Safranal	299	2M-H	1.48	4.77 × 10^8^	2.70 × 10^6^
	*Gamma*-Crocetin	117	M-3H	1.85	1.53 × 10^8^	6.28 × 10^7^
	Unknown	692		2.62	1.05 × 10^9^	1.17 × 10^8^
	Nonanoate	361	2M+FA^f^−H	5.08	5.92 × 10^8^	1.62 × 10^9^
	Carotene	535	M-H	5.24	3.41 × 10^8^	6.15 × 10^5^
	Unknown	702		5.40	8.56 × 10^5^	4.59 × 10^7^
	Crocusatin G	392	2M-H	5.41	1.09 × 10^9^	6.46 × 10^8^
	Unknown	753		5.43	1.84 × 10^6^	1.57 × 10^7^
	Unknown	389		5.72	1.52 × 10^8^	1.01 × 10^8^
	(all-*E*)-Crocetin	373	M+FA-H	5.91	6.29 × 10^6^	7.50 × 10^5^
	Picrocrocin	375	M+FA-H	5.95	1.53 × 10^9^	1.18 × 10^8^
	5-Hydroxymethyl-2-furancarboxaldehyde	377	3M-H	6.08	2.06 × 10^8^	2.66 × 10^7^
	Unknown	595		6.38	1.73 × 10^6^	5.06 × 10^8^
	Rhamnetin	948	3M-H	6.50	2.57 × 10^8^	1.32 × 10^7^
	Unknown	757		6.60	2.78 × 10^5^	4.99 × 10^8^
	*Beta*-Sitosterol	460	M+FA-H	6.92	1.53 × 10^7^	1.88 × 10^8^
	Unknown	346		7.30	1.63 × 10^8^	3.25 × 10^6^
	Unknown	368		7.56	1.43 × 10^8^	6.20 × 10^8^
	Unknown	371		10.33	5.50 × 10^7^	2.80 × 10^8^
	Norathyriol	565	2M+FA-H	10.98	1.17 × 10^8^	2.59 × 10^8^
	Riboflavin	357	M-H_2_O-H	12.70	4.66 × 10^7^	2.33 × 10^8^
(ESI)^+^	Unknown	87		2.01	3.06 × 10^8^	3.65 × 10^8^
	Isopimpinellin	105	M+H+2Na	2.18	5.00 × 10^9^	6.63 × 10^7^
	Unknown	116		2.46	2.37 × 10^8^	6.08 × 10^9^
	4H-Pyran-4-one-2, 3-dihydro-3, 5-dihydroxy-6-methyl	84	M+H+Na	2.57	1.64 × 10^8^	1.90 × 10^8^
	Osthol	104	M+3Na	2.62	6.16 × 10^8^	7.89 × 10^7^
	3, 5,5-Trimethyl-2-hydroxy-1, 4-cyclohexadion-2-ene	103	M+H+K	3.01	6.15 × 10^9^	2.77 × 10^8^
	2, 4, 4-Trimethyl-3-formyl-6-hydroxy-2, 5-cyclohexadien-1-one	82	M+3Na	3.68	2.72 × 10^8^	3.87 × 10^9^
	Unknown	119		9.34	3.45 × 10^7^	5.48 × 10^9^
	Methyl arachidate	125	M+H+2Na	13.63	2.73 × 10^8^	1.34 × 10^9^
	Unknown	154		14.07	5.44 × 10^8^	1.59 × 10^9^
	Kaempferol 3-*O*-alpha-L-rhamnofuranoside	167	M+3Na	14.24	7.65 × 10^7^	5.85 × 10^9^
	Isophorone	138	M+H	15.01	5.91 × 10^8^	2.07 × 10^9^
	Crocin 4	169	M+3H	15.40	4.82 × 10^9^	7.43 × 10^8^
	4H-Pyran-4-one-2, 3-dihydro-3, 5-dihydroxy-6-methyl	71	M+3Na	15.45	1.40 × 10^7^	1.31 × 10^9^

^a^The name of the identified chemical compounds. ^b^The extracted mass of chemical compounds. ^c^The identified adducts. ^d^The retention times of the elution peak maximum. ^e^The area under each of the resolved chromatographic profiles in C matrices by MCR-ALS. ^f^Formic acid.

**Table 3:** IC_50_ values of the stigmas ethanol extract against MDA-MB-231 and MCF-7 in monolayer (2D) and spheroid (3D) cultures.

Type of culture	IC_50_ (*μ*g/mL)			
	MDA-MB-231		MCF-7	
	24 hours	72 hours	24 hours	72 hours
Two-dimensional (2D) monolayers	97.54 ± 9.66^a,b^	69.06 ± 6.55^a,b^	131.44 ± 8.36^a,b^	78.70 ± 9.09^a,b^
Three-dimensional (3D) spheroid	325.13 ± 15.21^a,b^	155.45 ± 14.22^a,b^	>400	>400

The concentrations of the extract that could provide 50% cytotoxic activity (IC_50_) were calculated from the graph that plotted the inhibition percentage against different concentrations. ^a^IC_50_ values are significantly different between 24 and 72 Hours. ^b^The IC_50_ values are significantly different between 2D and 3D cultures.

## Data Availability

The research data used to support the findings of this study are included in the article.

## Supplementary Materials

Supplementary Materials Supplementary Figure S1: gas chromatogram of *C*. *cancellatus* subsp. damascenus stigma ethanol extract.
