# Supplementary figures and images for: Untargeted Metabolomics Analysis of Crocus cancellatus subsp. damascenus (Herb.) B. Mathew Stigmas and Their Anticarcinogenic Effect on Breast Cancer Cells
Source: Evid Based Complement Alternat Med. 2022 Aug 16;2022:3861783. doi: 10.1155/2022/3861783 (PMC9398734; doi:10.1155/2022/3861783)

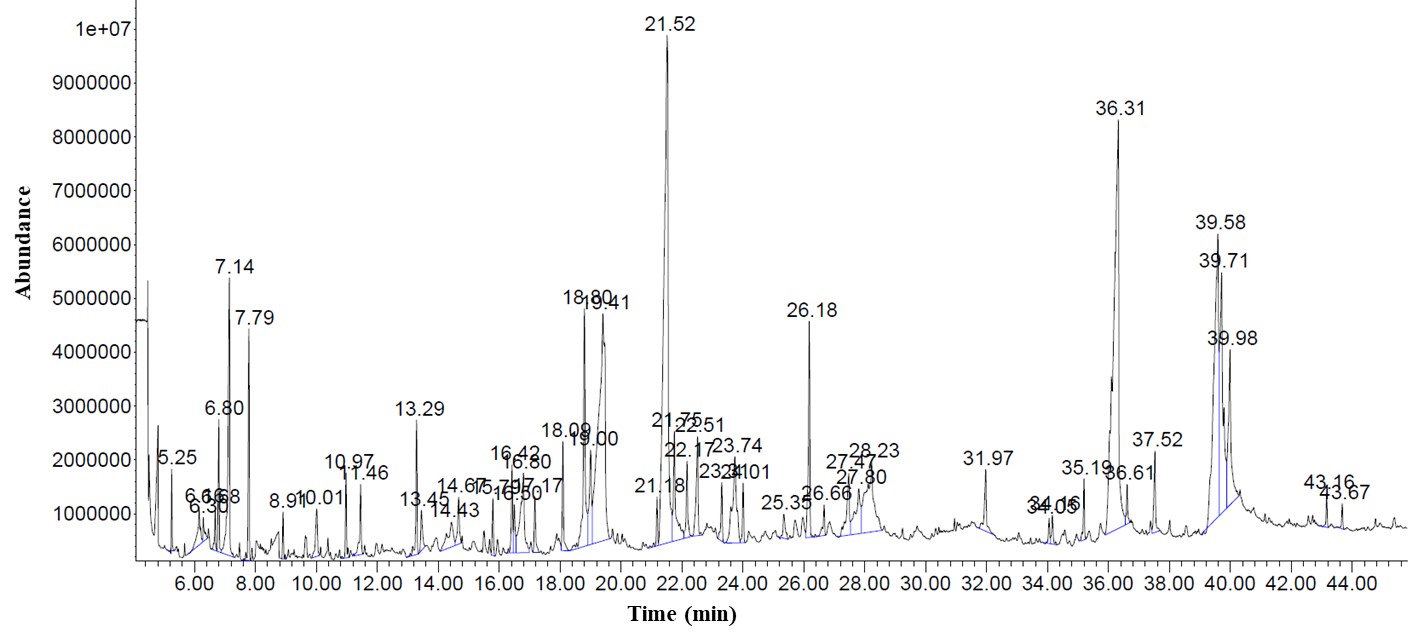


**Figure S1.** Gas chromatogram of *C. cancellatus* subsp. *damascenus* stigmas ethanol extract.

Supplement: Supplementary Materials — Supplementary Figure S1: gas chromatogram of C. cancellatus subsp. damascenus stigma ethanol extract. [file 3861783.f1.docx]
